# An increase in splenic volume after first-line immunotherapy is associated with worse PFS in patients with metastatic renal cell carcinoma

**DOI:** 10.1093/oncolo/oyaf397

**Published:** 2026-01-06

**Authors:** Gregory Palmateer, Ahmet Yildirim, Taylor Goodstein, Dattatraya Patil, Samay Patel, Shreyas Joshi, Vikram Narayan, Jacqueline T Brown, Bassel Nazha, Shahid S Ahmed, Jordan Ciuro, Bradley C Carthon, Omer Kucuk, Haydn Kissick, Kenneth Ogan, Mehmet A Bilen, Viraj A Master

**Affiliations:** Department of Urology, Emory University School of Medicine, Atlanta, GA 30322, United States; Department of Hematology and Oncology, Emory University School of Medicine, Atlanta, GA 30322, United States; Department of Urology, Emory University School of Medicine, Atlanta, GA 30322, United States; Department of Urology, Emory University School of Medicine, Atlanta, GA 30322, United States; Department of Urology, Emory University School of Medicine, Atlanta, GA 30322, United States; Department of Urology, Emory University School of Medicine, Atlanta, GA 30322, United States; Winship Cancer Institute of Emory University, Atlanta, GA 30322, USA; Department of Urology, Emory University School of Medicine, Atlanta, GA 30322, United States; Winship Cancer Institute of Emory University, Atlanta, GA 30322, USA; Department of Hematology and Oncology, Emory University School of Medicine, Atlanta, GA 30322, United States; Winship Cancer Institute of Emory University, Atlanta, GA 30322, USA; Department of Hematology and Oncology, Emory University School of Medicine, Atlanta, GA 30322, United States; Winship Cancer Institute of Emory University, Atlanta, GA 30322, USA; Piedmont Cancer Institute, Atlanta, GA 30322, United States; Department of Hematology and Oncology, Emory University School of Medicine, Atlanta, GA 30322, United States; Winship Cancer Institute of Emory University, Atlanta, GA 30322, USA; Department of Hematology and Oncology, Emory University School of Medicine, Atlanta, GA 30322, United States; Winship Cancer Institute of Emory University, Atlanta, GA 30322, USA; Department of Hematology and Oncology, Emory University School of Medicine, Atlanta, GA 30322, United States; Winship Cancer Institute of Emory University, Atlanta, GA 30322, USA; Department of Hematology and Oncology, Emory University School of Medicine, Atlanta, GA 30322, United States; Winship Cancer Institute of Emory University, Atlanta, GA 30322, USA; Department of Urology, Emory University School of Medicine, Atlanta, GA 30322, United States; Department of Urology, Emory University School of Medicine, Atlanta, GA 30322, United States; Winship Cancer Institute of Emory University, Atlanta, GA 30322, USA; Department of Hematology and Oncology, Emory University School of Medicine, Atlanta, GA 30322, United States; Winship Cancer Institute of Emory University, Atlanta, GA 30322, USA; Department of Urology, Emory University School of Medicine, Atlanta, GA 30322, United States; Winship Cancer Institute of Emory University, Atlanta, GA 30322, USA

**Keywords:** immunotherapy, immuno-oncology, radiologic biomarkers, splenic change, renal cell carcinoma

## Abstract

**Importance:**

Reliable prognostic markers for immune checkpoint inhibitor (ICI) response in metastatic renal cell carcinoma (mRCC) remain limited.

**Objective:**

To examine the impact of splenic volume change after ICI initiation on progression-free survival (PFS) and overall survival (OS) in patients with mRCC.

**Design:**

A retrospective cohort study reviewing data from 2015 to 2023.

**Setting:**

The Emory Kidney Cancer database (single-center academic instution).

**Participants:**

Patients with mRCC who underwent first-line ICI treatment and had available abdominal imaging 30 days before and 60-120 days after ICI initiation. A total of 109 patients met inclusion criteria.

**Exposure:**

Splenic volume change calculated as a percentage difference between baseline and follow-up imaging (median 2.8 months post-initiation) using a standardized formula, grouped into ≥10% increase and <10% increase.

**Main Outcomes and Measures:**

Differences in OS and PFS assessed using Kaplan–Meier curves and multivariable Cox hazards regression models.

**Results:**

A total of 109 patients met inclusion criteria. Median follow-up time was 25.2 months (IQR 11.2-41.5), during which there were 47 mortality events. Patients with a splenic volume increase ≥ 10% at a median 2.8 months after ICI initiation had worse 2-year PFS (28.5% vs 50.4%, *P* = .022) but not OS (69.4% vs 77.8%, *P* = .853) compared to patients with a < 10% increase in splenic volume. On multivariable analysis, a splenic volume increase ≥ 10% was independently associated with worse PFS (2.33 [95% CI 1.37-3.96], *P* = .002).

**Conclusions and Relevance:**

In patients with mRCC, a splenic volume increase ≥ 10% at a median of 2.8 months following ICI initiation is independently associated with worse survival compared to an < 10% increase. Monitoring splenic volume changes may serve as a cost-effective radiographic prognostic marker to guide treatment sequencing.

## Graphical Abstract

**Graphical Abstract:**
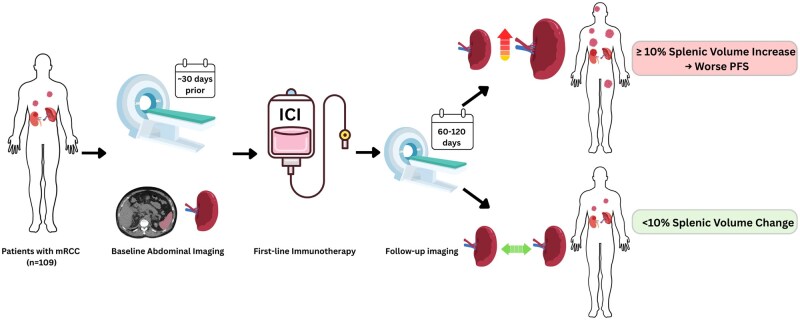

Implications for Practice In patients with metastatic renal cell carcinoma, reliable early prognostic markers for first-line immune checkpoint inhibitor therapy are needed. Our study found that a splenic volume increase of ≥10%, measured on routine follow-up imaging at a median of 2.8 months after starting treatment, is independently associated with worse progression-free survival. This finding suggests splenic volume change may serve as a cost-effective prognostic biomarker, potentially helping clinicians identify high-risk patients for closer monitoring and counseling.

## Introduction

Immuno-oncology agents have become the cornerstone of management in patients with metastatic renal cell carcinoma (mRCC). The modern standard of care for first-line treatment consists of immune checkpoint inhibitors (ICIs) targeting the programmed death-1 (PD-1) or programmed death-ligand 1 (PD-L1) pathways, combined with either tyrosine kinase inhibitors (TKIs) or cytotoxic T-lymphocyte-associated protein 4 (CTLA-4) inhibitors.^1–4^ While ICI doublet therapy has greatly improved survival outcomes over prior first-line options, the objective response rates of these regimens range from 42% to 71%, and complete response rates (CR) range from 9% to 16%.^5^ However, we currently lack reliable predictive factors to distinguish, either before initiating or early in treatment, responders from non-responders.^6^ Developing tools to address this gap is essential for optimizing treatment strategies.

The spleen is a secondary lymphoid organ that is responsible for filtering blood and mounting an immune response against pathogens. It plays a central role in both innate and adaptive immunity, facilitating antigen presentation, immune cell activation, and cytokine production.^7^^,^^8^ The spleen harbors diverse immune cell populations, including dendritic cells, T cells, B cells, and myeloid-derived suppressor cells (MDSCs), which help regulate immune responses. Among these, MDSCs are of particular interest due to their immunosuppressive properties that influence cancer progression and treatment resistance.^9–11^ Given its regulatory role in immune homeostasis, changes in splenic architecture and function may reflect systemic immune alterations in response to ICI cancer therapies, and serve as a trackable biomarker.

The interaction between ICIs and the spleen is an area of growing interest in oncology. Previous studies suggest that ICIs can induce dynamic changes in immune cell populations, including those residing in the spleen.^12^ MDSCs, which accumulate in both the spleen and peripheral blood of cancer patients, are known to suppress T cell proliferation and function, contributing to neoplasm immune evasion.^13^ Changes in splenic volume have been observed in patients undergoing immunotherapy, raising the question of whether splenic size alterations could serve as a surrogate marker for immune response to systemic treatment and, consequently, disease prognosis.^14–18^ Specifically, changes in splenic volume following ICI initiation have been reported in malignancies, such as melanoma and non-small-cell lung cancer, and volumetric increases were found to be associated with worse progression-free survival (PFS) and overall survival (OS).^14–18^ The prognostic utility of splenic change in patients with mRCC receiving ICI is largely unstudied. Recently, a 2023 study found that an increase in splenic volume after initiation of second-line nivolumab was associated with significantly worse OS and PFS in 45 patients with mRCC.^15^ To the best of our knowledge, no study has examined the relationship between splenic volume change and outcomes in mRCC patients receiving first-line immunotherapy. Thus, the purpose of our study was to examine whether changes in splenic volume can serve as a prognostic biomarker for treatment response in patients with mRCC treated with ICIs in the first-line setting.

## Methods

### Patient selection

We retrospectively reviewed the Emory Kidney Cancer database for patients with mRCC who underwent any first-line ICI between 2015 and 2023 under an IRB-approved protocol. Patients with available abdominal computerized tomography (CT) or magnetic resonance (MR) imaging within 30 days before and 60-120 days after ICI initiation were included. Patients who received ICI in the neoadjuvant or adjuvant settings, asplenic patients, as well as patients with a history of autoimmune conditions, chronic liver disease, or infections known to alter spleen size (eg, hepatitis B/C, tuberculosis, brucellosis, or candidemia) were excluded. Patient demographics, oncologic/treatment characteristics, and laboratory values were collected from the database. Baseline and follow-up splenic volume was calculated using the formula 30 + (0.58 × width × length × thickness), and the change was reported as a percentage difference. Previous publications have shown that this formula correlates well with actual splenic volume measurements.^19^^,^^20^ Patients were then categorized into 2 categorical groups: ≥ 10% increase in splenic volume, < 10% increase in splenic volume. Immune-related adverse events (irAE) were identified using the Common Terminology Criteria for Adverse Events version 5.0, with major events being defined as grade 3 or higher. All patients were followed until death or until follow-up by August 2024. Patients with no further follow-up were censored at the time of the last clinical contact. Primary outcomes were OS and PFS. OS was defined as time to death attributed to any cause, while PFS was defined as time to the occurrence of disease progression or death. This was cross-referenced utilizing the electronic medical record, the state of Georgia death registry, and the national (United States) death index. Secondary outcome was the development of irAE and associated factors.

### Statistical analysis

Continuous variables were calculated as median values with interquartile ranges (IQR), and categorical variables as counts and percentages. ANOVA and chi-square tests were used to compare differences between groups. Kaplan–Meier curves were used to estimate 2-year OS and PFS rates. The association between the percent change of splenic volume with OS and PFS after first-line ICI was determined utilizing a multivariable Cox proportional hazards model. Additionally, multivariable logistic regression was used to determine factors associated with the development of irAE. Variables including patient factors, oncologic treatment details, and laboratory results were included as potential confounders in multivariable analyses. Backward selection was performed to arrive at the final models. Collinearity and interaction were assessed, and Harrell’s concordance statistic estimates were calculated for each model. All statistical tests were 2-sided with type 1 error set at 0.05. All analyses were performed using SAS version 9.4.

## Results

A total of 109 patients met inclusion criteria. The median follow-up time was 25.2 months (IQR 11.2-41.5), during which there were 47 mortality events. Follow-up imaging was obtained a median of 2.8 months (IQR 2.5-3 months) after IO initiation. There were 36 (33%) patients with a ≥ 10% increase in splenic volume following immunotherapy initiation. Clear cell histology was observed in 73.4% of the cohort. Black patients were significantly more likely to present with non-clear cell histology compared to white patients (57.9% vs 26.6%, *P* = .003). Differences in patient and oncologic characteristics between patients with and without a ≥ 10% increase in splenic volume are displayed in Table 1. Most patients received singlet or doublet ICI therapy (65.14%); there was no significant difference between groups. Additionally, there was no significant difference in age, sex, race, obesity, smoking status, hypertension, diabetes mellitus, count of metastatic sites, type of prior kidney surgery, histology, type of systemic therapy, IMDC risk score, and immune-related adverse events in patients with and without a ≥ 10% increase in splenic volume. Patients with a ≥ 10% increase in splenic volume were significantly more likely to have elevated median neutrophil-lymphocyte ratio (NLR; 3.4 vs 2.8, *P* = .018) and platelet-lymphocyte ratio (PLR; 197.3 vs 170.9, *P* = .004) compared to patients without a ≥ 10% increase in splenic volume. They were also observed to have a higher median percent change in NLR following ICI initiation compared to patients without a ≥ 10% increase in splenic volume (9% vs −21.7%, *P* = .035).

**Table 1.** Patient characteristics.

		Splenic volume increase ≥ 10%			
Covariables		No *N* = 73	Yes *N* = 36	Total *N* = 109	*P*-value
**Age > 65 years**		34 (46.6)	21 (58.3)	55 (50.46)	.248
**Male**		55 (75.3)	23 (63.9)	78 (71.56)	.213
**Race**					.163
	Black	16 (21.9)	3 (8.3)	19 (17.43)	
	Other	6 (8.2)	2 (5.6)	8 (7.34)	
	White	51 (69.9)	31 (86.1)	82 (75.23)	
**ECOG PS ≥ 1**		48 (65.8)	22 (61.1)	70 (64.22)	.634
**BMI ≥ 30**		22 (30.1)	12 (33.3)	34 (31.19)	.735
**Smoking status**					.300
	Current	12 (16.4)	3 (8.3)	15 (13.76)	
	Former	21 (28.8)	8 (22.2)	29 (26.61)	
	Never	40 (54.8)	25 (69.4)	65 (59.63)	
**HTN**		47 (64.4)	27 (75)	74 (67.89)	.264
**DM**		22 (30.1)	10 (27.8)	32 (29.36)	.799
**Count of metastatic disease sites**					.536
	0-1	19 (26)	11 (30.6)	30 (27.52)	
	2	26 (35.6)	9 (25)	35 (32.11)	
	3 or more	28 (38.4)	16 (44.4)	44 (40.37)	
**Kidney surgery**					.095
	Ablation	0 (0)	2 (5.6)	2 (1.83)	
	None	20 (27.4)	8 (22.2)	28 (25.69)	
	Partial	4 (5.5)	0 (0)	4 (3.67)	
	Radical	49 (67.1)	26 (72.2)	75 (68.81)	
**Clear cell histology**		52 (71.2)	28 (77.8)	80 (73.39)	.467
**Systemic therapy type**					.814
	ICI + TKI	26 (35.6)	12 (33.3)	38 (34.86)	
	ICI singlet or doublet	47 (64.4)	24 (66.7)	71 (65.14)	
**IMDC risk**					.822
	Favorable-intermediate risk	52 (72.2)	26 (74.3)	78 (72.9)	
	Poor risk	20 (27.8)	9 (25.7)	29 (27.1)	
**Immune-related AE**					.864
	Major	7 (9.6)	4 (11.1)	11 (10.09)	
	Minor	40 (54.8)	21 (58.3)	61 (55.96)	
	None	26 (35.6)	11 (30.6)	37 (33.94)	
**6 week neutrophil to lymphocyte ratio** ^a^		2.8 (1.8-4.6)	3.4 (2.1-6.6)	3 (1.9-5.2)	**.018**
**6-week monocyte to lymphocyte ratio** ^a^		0.4 (0.3-0.6)	0.4 (0.3-0.7)	0.4 (0.3-0.6)	.093
**6-week platelet to lymphocyte ratio** ^a^		170.9 (125-248.2)	197.3 (128.8-417.9)	177.2 (125.9-274.2)	**.004**
**NLR percent change from baseline** ^a^		−21.7 (−42.1-5.6)	9 (−21.4-50)	−15 (−35.8-20)	**.047**

Results reported number (%). Parametric *P*-value by ANOVA for numerical and chi-square test for categorical covariates. Significant *P*-values (<.05) are bolded.

Abbreviations: AE, adverse events; BMI, body mass index; ECOG PS, Eastern Cooperative Oncology Group performance status; HTN, hypertension; ICI, immune checkpoint inhibitor; IMDC, International Metastatic Renal Cell Carcinoma Database Consortium; NLR, neutrophil to lymphocyte ratio; TKI, tyrosine kinase inhibitor.

a Median (interquartile range).

**Figure 1.**
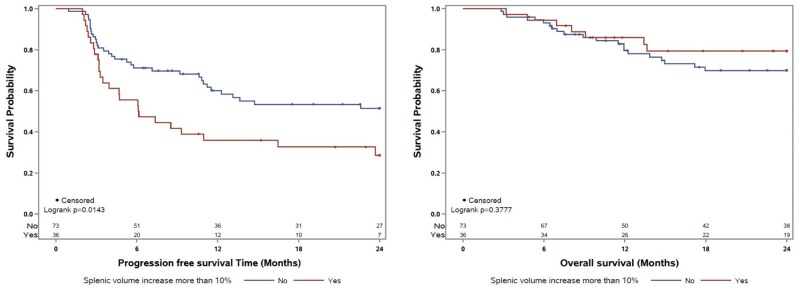
Kaplan–Meier survival curves for overall survival and progression-free survival in patients with metastatic renal cell carcinoma receiving first-line immunotherapy.

On Kaplan–Meier analysis, patients with a ≥ 10% increase in splenic volume at a median 2.8 months after ICI initiation had significantly worse 2-year PFS (28.5% vs 50.4%, *P* = .022), but not OS (69.4% vs 77.8%, *P* = .853) compared to patients with a < 10% increase in splenic volume (Figure 1; Supplementary Figure 1). On multivariable analysis, a ≥ 10% increase in splenic volume was independently associated with worse PFS (2.33 [95% CI 1.37-3.96], *P* = .002; Table 2). Additionally, an increase in NLR at 6 weeks from baseline was also significantly associated with worse PFS (HR 1.79 [95% CI 1.03-3.13], *P* = .040). Grade 1-2 irAEs were found to be associated with longer PFS (HR 0.57 [95% CI 0.32-0.99], *P* = .047). An increase in splenic volume was dropped from the final multivariable analysis assessing patient OS due to non-significance on backward selection.

**Table 2.** Multivariable Cox Hazard model for progression-free survival among patients with metastatic renal cell carcinoma receiving first-line immunotherapy.

Covariates		*N* (%)	HR (95% CI)	*P*-value
**Change in splenic volume ≥ 10%**		36 (33)	2.33 (1.37-3.96)	**.002**
**HTN**		74 (67.9)	0.57 (0.33-0.98)	**.042**
**ccRCC**		80 (73.4)	0.60 (0.33-1.10)	.101
**IMDC Score**				
	Poor risk	29 (27.1)	1.39 (0.76-2.54)	.287
	Favorable-intermediate risk	78 (72.9)	Ref	
**Immune-related AE**				
	Major	11 (10.1)	0.64 (0.28-1.47)	.293
	Minor	61 (56)	0.57 (0.32-0.99)	**.047**
	None	37 (33.9)	Ref	
**Any increase in NLR following treatment^a^**			1.79 (1.03-3.13)	**.040**

Abbreviations: AE, adverse events; ccRCC, clear cell renal cell carcinoma; CI, confidence interval; HR, hazard ratio; HTN, hypertension; IMDC, International Metastatic RCC Database Consortium; NLR, neutrophil-lymphocyte ratio.

a Continuous variable. Number of observations in the original data set = 109. Number of observations used = 104. Harrell’s concordance statistic estimate = 0.6956. Variables which dropped off during model backward selection included Sex, Race, Eastern Cooperative Oncology Group (ECOG) Performance Status, Obesity, Diabetes Mellitus, Type of First-line Treatment, and Count of Metastatic Sites.

A total of 72 (66.1%) patients experienced an irAE following immunotherapy treatment. Among these, 11 (15.3%) patients had a grade 3 or higher adverse event. On logistic regression, black race was found to be protective (HR 0.23 [95% CI 0.06-0.87], *P* = .030) against experiencing an irAE (Table 3). No other factors were significantly associated with the development of an irAE.

**Table 3.** Logistic regression for factors associated with immune-related adverse events among patients with metastatic renal cell carcinoma receiving first-line immunotherapy.

Covariables		Hazard ratio (95% CI)	*P*-value
**Increase in splenic volume ≥ 10%**		1.03 (0.38-2.82)	.954
**Age ≥ 65**		0.47 (0.19-1.19)	.110
**Female sex**		2.81 (0.87-9.04)	.084
**Race**			
	Black	0.23 (0.06-0.87)	**.030**
	Other	1.07 (0.15-7.82)	.946
	White	Ref	
**BMI ≥ 30 kg/m^2^**		2.43 (0.91-6.49)	.077
**Clear cell histology**		0.36 (0.10-1.28)	.116
**Type of first-line treatment**			
	ICI singlet or doublet	0.53 (0.19-1.51)	.237
	ICI + TKI	Ref	
**HTN**		1.72 (0.63-4.74)	.293

Number of observations in the original data set = 109 Number of observations used = 95. Variables dropped from the model in backward selection: Age > 65 years, Eastern Cooperative Oncology Group (ECOG) Performance Status, Diabetes Mellitus, Count of metastatic sites, type of first-line treatment, International Metastatic Renal Cell Carcinoma Database Consortium (IMDC) Risk score, and change in NLR after treatment.

Abbreviations: BMI, body mass index; CI, confidence interval; HTN, hypertension; ICI, immune checkpoint inhibitor; TKI, tyrosine kinase inhibitor.

## Discussion

In this study, we demonstrate that an increase in splenic volume ≥ 10% a median of 2.8 months following the initiation of first-line ICI is independently associated with worse PFS in patients with mRCC. An increase in NLR from baseline to 6 weeks post-treatment was also correlated with shorter PFS, a finding that falls outside our hypothesis but is related to the role that systemic inflammation plays in ICI treatment outcomes.

The spleen is a key immunologic organ involved in innate and adaptive immune regulation.^8^ It serves as a major reservoir for MDSCs, a heterogeneous population of immunosuppressive cells that expand in response to cancer and chronic inflammation.^21^ MDSCs are known to inhibit CD4+ and CD8+ T-cell proliferation and function, ultimately dampening antitumor immunity and promoting resistance to ICIs.^11^^,^^22^^,^^23^ MDSC can be broken down into 2 subtypes: polymorphonuclear or granulocytic MDSCs (CD15+) and monocytic MDSC (CD14+).^21^ Splenectomy specimens from patients with pancreatic cancer have been reported to have increased CD15+ MDSCs within the spleen compared to patients with benign pancreatic cysts.^21^ No studies have directly correlated MDSC levels in the spleen to those within the tumor microenvironment; however, elevated levels of MDSCs in both the spleen and tumor microenvironment have been shown to correlate with elevated levels of MDSCs in peripheral circulation.^24^^,^^25^ Although the precise mechanisms are not yet fully understood, increases in splenic volume following immunotherapy have been well described in prior studies.^14–18^ Increases in splenic volume may correspond to an increase in number and activity of MDSCs within the spleen and tumor microenvironment, which could contribute to systemic immunosuppression, poorer treatment response, and shorter survival.^26^^,^^27^ Taken together, these findings raise the possibility that an increase in splenic volume may serve as an indirect marker of immune dysregulation following ICI initiation.

Changes in splenic volume following initiation of ICIs have shown prognostic relevance across multiple malignancies, though only one prior study has examined this in mRCC. In that study, Aslan et al. found that a ≥ 10% increase in splenic volume after second- or third-line nivolumab was independently associated with worse progression-free and overall survival. In our cohort of patients receiving first-line ICI, we similarly observed an association between ≥10% splenic volume increase and shorter PFS, though not overall survival. Differences in treatment line, patient age, IMDC risk, and histology—our cohort had more non-clear cell tumors and poorer risk features—may explain the discrepancy. Broader literature on this topic across melanoma, non-small-cell lung cancer, urothelial, and hepatocellular cancers has yielded mixed results, with splenic enlargement linked to worse outcomes in some studies, no association in others, and improved survival in a few **(**Table 4**)**. These inconsistencies likely reflect heterogeneity in treatment setting, study design, and volume change definitions. Thresholds for “clinically meaningful” change varied from any directional shift to absolute or proportional increases, limiting comparability. Together, these findings highlight the need for standardized criteria and methodology when evaluating splenic volume as a biomarker in ICI-treated populations.

**Table 4.** Summary of studies evaluating splenic volume changes following immune checkpoint inhibitor therapy and survival outcomes.

Study (Author, Year)	Cancer type	*n*	Received treatment	Splenic volume change definition	Key findings
**Susok et al. (2021)^16^**	Melanoma	49	Nivolumab, Ipilimumab+ Nivolumab	Continuous; median change	Increased spleen volume was observed at 3 months following ICI initiation, but it was not significantly associated with survival measures
**Galland et al. (2021)^14^**	NSCLC	276	PD-1/PD-L1 and/or CTLA-4 inhibitors	Continuous; no predefined cut-off	Spleen volume change correlated with shorter OS (HR 2.10, 95% CI [1.1–3.8], *P* = .01), but not significantly with PFS (HR 1.3, 95% CI [0.8–2.1], *P* = .2)
**Castagnoli et al. (2022)^46^**	NSCLC	70	Pembrolizumab, Pembrolizumab + Chemo	Continuous; no predefined cut-off	No significant difference in spleen volume change between patients who benefited from treatment and those who did not
**Müller et al. (2022)^17^**	Hepatocellular Carcinoma	50	Pembrolizumab, Atezolizumab + Bevacizumab, Nivolumab	Continuous; no predefined cut-off	Post-treatment increase in spleen volume was not associated with OS (7.0 vs 8.5 months, *P* = .73)
**Aslan et al. (2023)^15^**	Renal cell carcinoma	45	Nivolumab	> 10 % relative increase	Increased spleen volume was associated with shorter OS (HR 2.5, 95% CI [1–6], *P* = .048) and PFS (HR 2.1, 95% CI [1–4], *P* = .04)
**Duwe et al. (2023)^47^**	Urothelial and renal cell carcinoma	35 + 30	PD-1/PD-L1 and/or CTLA-4 inhibitors	Continuous; no predefined cut-off	No significant difference in OS between patients with increased vs. decreased spleen volume (24.0 vs 22.0 months, *P* = .643)
**Chen et al. (2024)^48^**	Hepatocellular carcinoma	143	PD-1 and/or CTLA-4 inhibitors, PD-1 + Antiangiogenic Therapy	Continuous; no predefined cut-off	Increased spleen volume following treatment was associated with longer PFS (HR 0.51, 95% CI [0.30–0.87], *P* = .014)
**Hatanaka et al. (2024)^29^**	Hepatocellular carcinoma	164	Atezolizumab + Bevacizumab	≥ 25 cm³ absolute change	Spleen volume change was not significantly associated with mOS (NR vs 24.5 months, *P* = .7) or mPFS (7.8 vs 6.6 months, *P* = .3)
**Mo et al. (2024)^28^**	Hepatocellular carcinoma	168	Atezolizumab + Bevacizumab	≥ 20 cm³ absolute change	Change in spleen volume was significantly associated with shorter OS (HR 4.20, 95% CI [1.40–12.57], p = 0.01)

Abbreviations: Chemo, chemotherapy; CI, confidence interval; Continuous, continuous variable; CTLA-4, cytotoxic T-lymphocyte-associated protein 4; HR, hazard ratio; ICI, immune checkpoint inhibitor; mOS, median overall survival; mPFS, median progression-free survival; NSCLC, non-small-cell lung cancer; OS, overall survival; PD-1, programmed cell death protein 1; PD-L1, programmed cell death ligand 1; PFS, progression-free survival.

RCC is an inherently immunogenic malignancy with a unique tumor microenvironment that differentiates it from other malignancies.^30^ The tumor microenvironment in RCC is characterized by a highly heterogeneous array of tumor-infiltrating lymphocytes whose complex interactions contribute to immune dysfunction and tumor progression.^31^^,^^32^ CD8+ T-cell tumor infiltration is associated with poor survival in RCC but is a favorable prognostic factor in other cancers such as non-small-cell lung cancer and colorectal cancer.^33–35^ The immunologic mechanisms within the RCC landscape are complex and not yet fully understood. Additional studies are needed to better characterize how increases in splenic volume, potentially reflecting MDSC proliferation, may influence the tumor microenvironment and treatment response in RCC.

Prior studies have demonstrated NLR may also serve as a proxy for dynamic changes in MDSC levels.^36^^,^^37^ Polymorphonuclear MDSCs possess similar phenotypic traits which make them indistinguishable from neutrophils on standard complete blood count measurements, and can lead to elevated levels of measured neutrophils. Additionally, MDSCs are also well known to suppress lymphocyte proliferation and activation. Consequently, elevations in MDSC levels frequently lead to corresponding elevations in NLR. Patients with a ≥ 10% increase in splenic volume in our study were significantly more likely to have an elevated NLR 6 weeks following ICI initiation compared to those without a < 10% increase. On multivariable analysis, the proportional increase in NLR from baseline to 6 weeks following ICI initiation was significantly associated with worse PFS, but not OS. Elevation in NLR following ICI treatment has been associated with both worse OS and PFS in patients with solid tumors.^38^^,^^39^ As discussed above, no record of subsequent treatment lines and cohort heterogeneity may also explain why change in NLR was dropped from the overall survival model during backward selection in our cohort. Future studies are needed to clarify whether an increase in splenic volume has prognostic value in overall survival among patients with mRCC.

A ≥ 10% increase in splenic volume was not predictive of experiencing an irAE. Black patients were significantly less likely to experience an irAE compared to white patients. This pattern has also been described in larger studies where black patients are at decreased risk for all-grade irAE, irAE requiring treatment with systemic steroids, or ICI discontinuation.^40^ It appears treatment toxicity is not entirely bad, however. ICI treatment toxicity can be indicative of immune system activation and has been associated with improved survival.^41^^,^^42^ Similar results were seen in our cohort, where experiencing a minor irAE was significantly associated with improved PFS. Prior studies have reported non-white patients have worse ICI-related survival compared to white patients; however, these have included non-clear cell RCC subtypes where ICI efficacy remains unclear. It is well understood non-white patients are more likely to have non-clear cell histology RCC compared to white patients.^43^^,^^44^ Studies which excluded patients with non-clear cell histology found no significant difference in PFS according to race in patients who received ICI therapy.^45^ Although we lacked the power to assess differences in ICI-related survival according to race, clear cell histology was associated with improved PFS, though it did not reach statistical significance.

Our study is not without limitations. As a retrospective single-institutional study, we were unable to control for potential confounding variables such as type and duration of first-line immunotherapy received. While the correlation between NLR and splenic change supports the underlying role of MDSCs, they are crude proxies to complex and dynamic immunological changes in response to immunotherapy. Future studies should look to validate our results and explore how splenic change could potentially inform treatment decisions following first-line immunotherapy.

## Conclusions

In patients with mRCC, an increase in splenic volume ≥ 10% at a median 2.8 months after first-line ICI was independently associated with worse PFS but not OS. Patients with a ≥ 10% increase in splenic volume were also more likely to have an increase in NLR following immunotherapy initiation, which was also significantly associated with worse PFS. Increases in splenic volume may serve as a cost-effective tractable biomarker for patients receiving first-line immunotherapy for mRCC.

## Supplementary Material

oyaf397_Supplementary_Data

## Data Availability

The data supporting the findings of this study are not publicly available due to HIPAA regulations and the need to protect patient confidentiality.
