# Supplementary material for: An increase in splenic volume after first-line immunotherapy is associated with worse PFS in patients with metastatic renal cell carcinoma
Source: Oncologist. 2026 Jan 6;31(2):oyaf397. doi: 10.1093/oncolo/oyaf397 (PMC12828282; doi:10.1093/oncolo/oyaf397)

**Supplementary Figure 1.** Kaplan-Meier survival curves for (A) overall survival and (B) progression-free survival in patients with metastatic renal cell carcinoma receiving first-line immunotherapy

**A** **B**
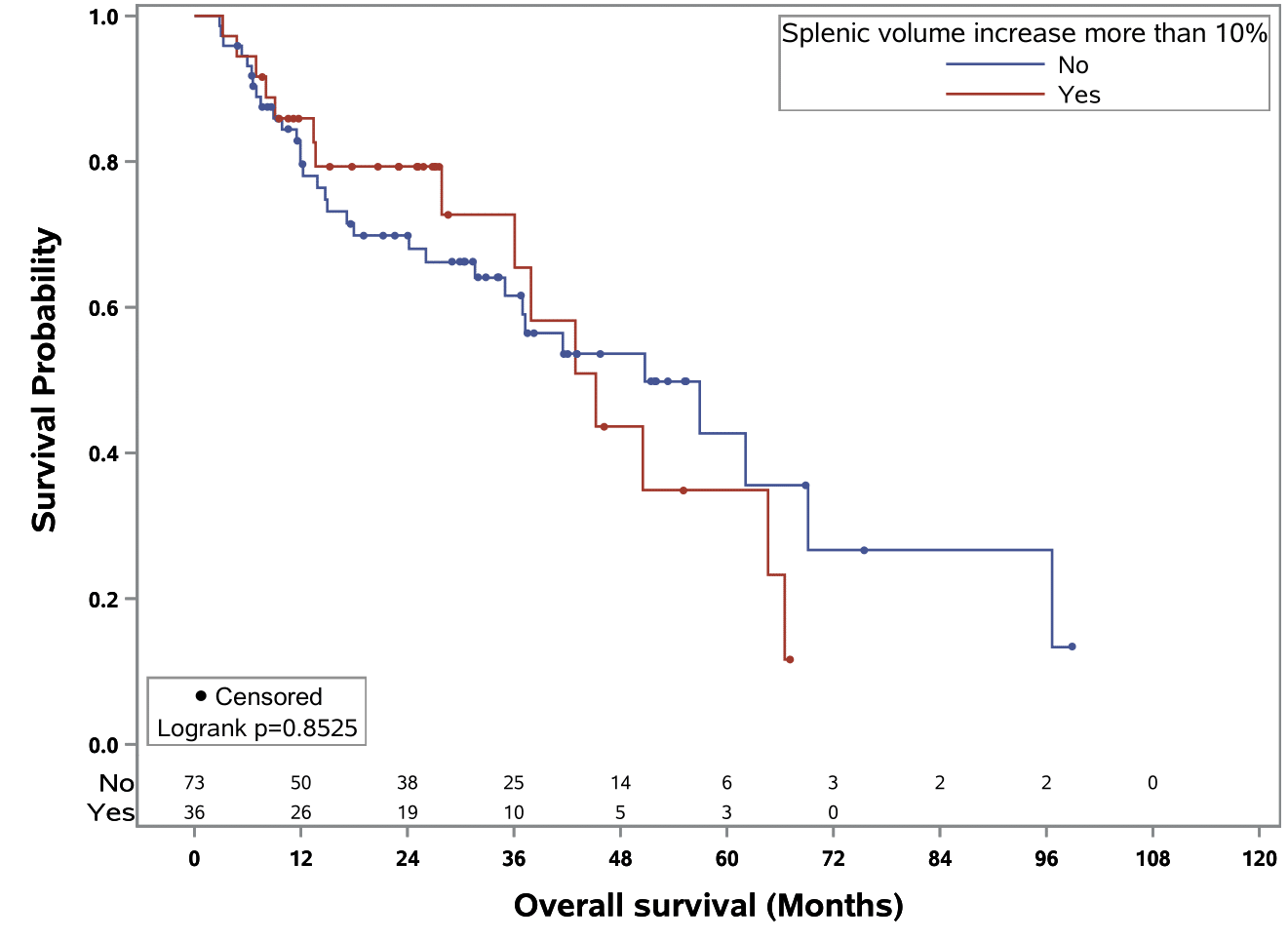

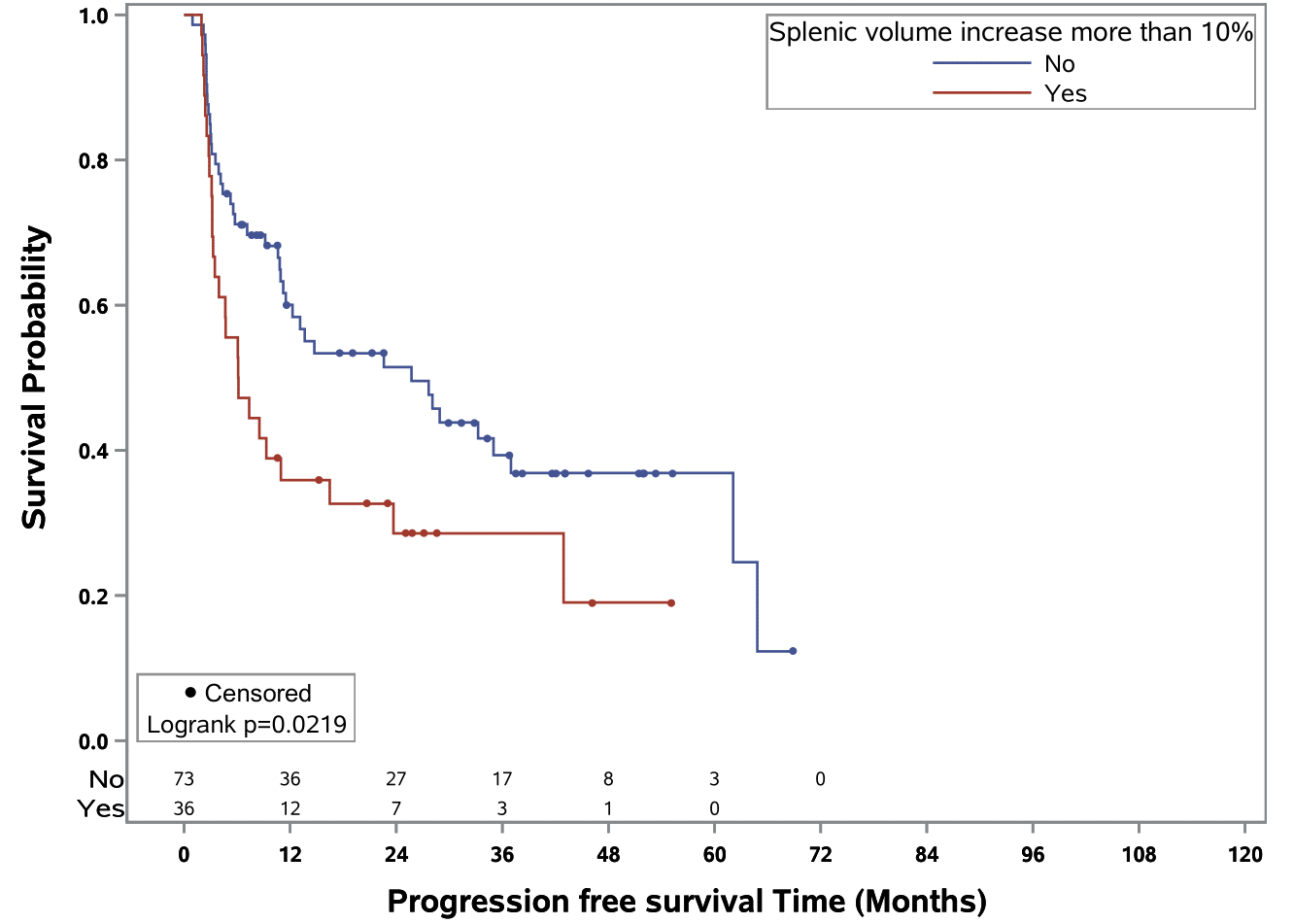

Supplement: oyaf397_Supplementary_Data [file oyaf397_supplementary_data.docx]
